# Editorial Change

**Published:** 1857-04

**Authors:** 


*Editorial Change.*

Dr. Goadby has retired from the post of Senior Editor of the *Medical Independent*, published at Detroit, Michigan. It is announced, however, that he will continue to be a contributor to its columns.

